# Opioid peptides in autism spectrum disorder and gluten-free casein-free diet as a therapeutic approach

**DOI:** 10.1007/s11011-026-01789-w

**Published:** 2026-01-31

**Authors:** Elif Öztürk, Nazlı Nur Aslan Çi̇n, Ali Cansu, Aslı Akyol

**Affiliations:** 1 Faculty of Health Sciences, Department of Nutrition and Dietetics, Karadeniz Technical University, Farabi Street No:88, Trabzon, Turkey; 2 Faculty of Medicine, Department of Pediatric Diseases, Karadeniz Technical University, Trabzon, Turkey; 3 Faculty of Health Sciences, Department of Nutrition and Dietetics, Hacettepe University, Ankara, Turkey

**Keywords:** Autism spectrum disorder, Gluteomorphin, Caseomorphin, Gluten-free casein-free diet, Child health

## Abstract

According to the Diagnostic and Statistical Manual of Mental Disorders, Fifth Edition, Autism Spectrum Disorder (ASD) is characterized by persistent difficulties in social communication and interaction, together with restricted and repetitive behaviors, interests, or activities. The diagnosis of ASD currently relies on comprehensive assessments of developmental history and behavioral patterns, as there are no validated laboratory tests for a definitive clinical diagnosis. While evidence-based interventions are largely restricted to educational and behavioral programs, many individuals with ASD and their caregivers explore complementary approaches, including dietary interventions. Among these, the gluten-free, casein-free (GFCF) diet is one of the most frequently adopted strategies. A leading hypothesis posits that those increased concentrations of opioid peptides such as gluteomorphin and caseomorphin derived from the incomplete digestion of gluten and casein may contribute to the severity of ASD symptoms. It is further suggested that eliminating these dietary proteins could reduce opioid peptide concentrations in biofluids and improve clinical outcomes. The present systematic review was created by reviewers who searched PubMed, Web of Science, and Scopus databases, covering the period from January 1980 to March 2025. The search strategy combined standardized keywords and Medical Subject Headings terms. The search strategy included a combination of keywords commonly used in the literature to represent ASD, opioid peptides, and GFCF diets. A systematic literature search was carried out on PubMed and Web of Science and a total of 17 articles were included. Although preliminary findings from clinical and laboratory studies are promising, conclusive evidence regarding the efficacy of the GFCF diet remains lacking. This review aims to synthesize current findings on the relationship between opioid peptides and ASD, with a particular focus on the neurological effects of food-derived peptides and their potential role in therapeutic dietary interventions.

## Introduction

According to the Diagnostic and Statistical Manual of Mental Disorders, Fifth Edition (DSM-5), Autism Spectrum Disorder (ASD) is characterized by persistent difficulties in social communication and interaction, together with restricted and repetitive behaviors, interests, or activities. These include challenges in social reciprocity, nonverbal communication, and relationship skills, as well as repetitive movements or speech, a preference for sameness and routines, intense or narrow interests, and unusual responses to sensory stimuli (APA 2013). Data from the Centers for Disease Control and Prevention’s (CDC) Autism and Developmental Disabilities Monitoring Network indicate a rising prevalence of ASD, with current estimates suggesting that approximately 1 in 31 children in the United States are affected (Shaw et al. 2025).

A growing body of evidence suggests that various environmental factors influencing early neurodevelopment contribute to the etiological heterogeneity of ASD. Among these factors, dietary components, alterations in gastrointestinal microbiota, and autoimmune mechanisms have been proposed as potential contributors to ASD pathogenesis (Shaw et al. 2025). Increasing attention has been directed toward the role of diet in both the onset and management of ASD symptoms. Specifically, dietary peptides such as those derived from gluten and casein have been implicated in the modulation of neurobehavioral outcomes through their effects on neurotransmitter systems and neural circuitry (Fetissov et al. 2019; Zurawicz et al. 2013). These peptides, commonly found in gluten- and casein-containing foods, may influence brain function and behavior, particularly in individuals with increased sensitivity or altered metabolic processing (Baspinar and Yardimci 2020; Hunter et al. 2003). Opioid-like peptides derived from food proteins are thought to possess significant biological activity. The hypothesized association between these peptides and ASD is based on the premise that certain individuals with ASD have impairments in peptide digestion and metabolism, leading to systemic accumulation. This accumulation may interact with central neurotransmitter systems, thereby contributing to the characteristic behavioral and cognitive symptoms of ASD (DSM-5, 2013; Zurawicz et al. 2013). Supporting this hypothesis, studies have demonstrated that in individuals with increased intestinal permeability, opioid peptides derived from gluten and casein can enter systemic circulation and potentially enhance opioid activity in the brain (Cass et al. 2008; Pellissier et al. 2018; Rueda-Ruzafa et al. 2020; Saxena et al. 2018).

**Fig. 1:**
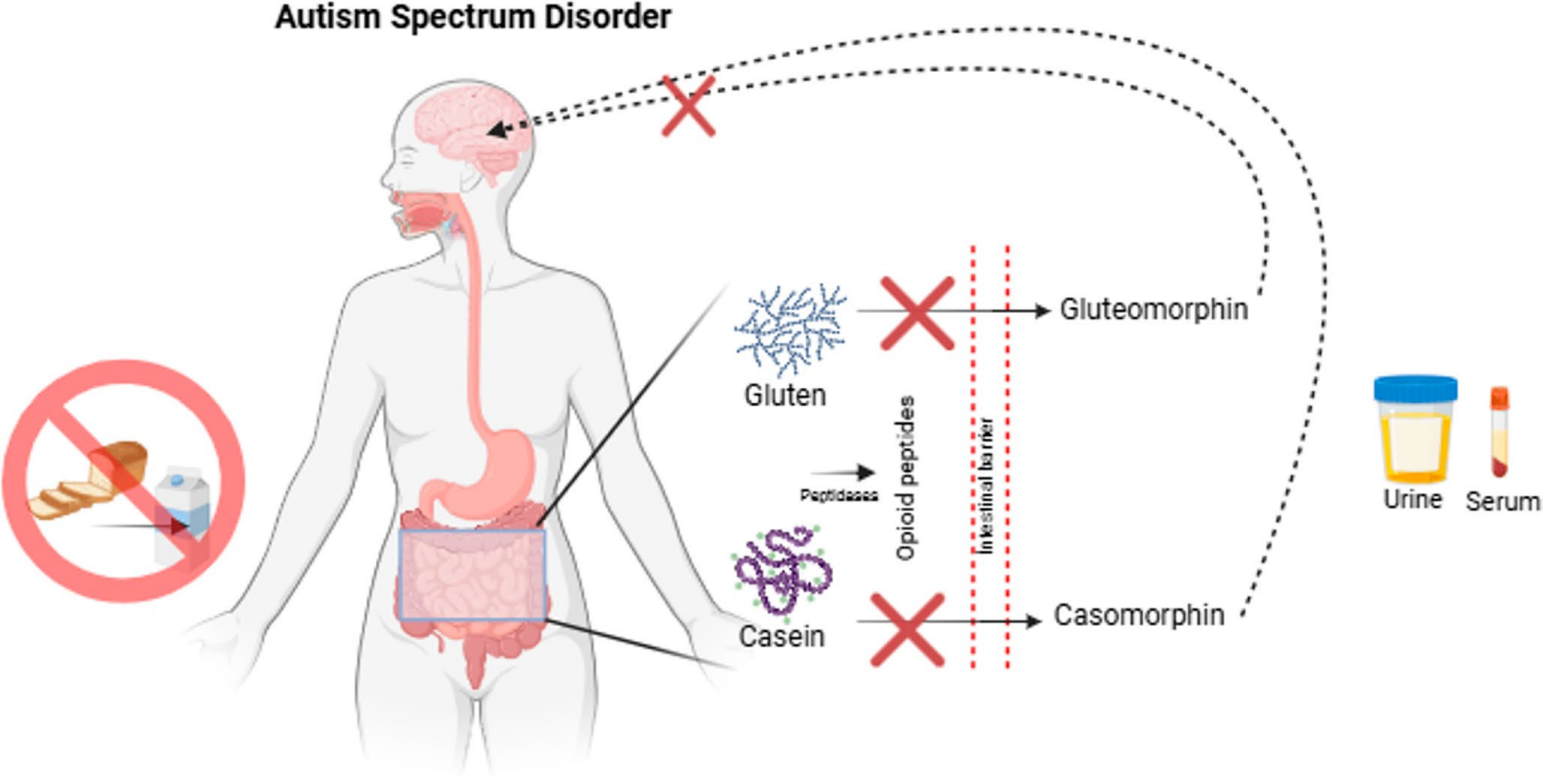
Graphical abstract. Consumption of a gluten-free, casein-free diet may decrease the concentration of opioid peptides such as gluteomorphin and caseomorphin in bio-fluids such as blood and urine. This may reduce the negative effects of opioid peptides on the brain

This review examines the potential effects of opioid peptide concentrations in individuals with ASD and specifically focuses on the regulatory roles of food-derived peptides, such as gluten and casein, on neurological functions and their significance in the treatment process (Fig. 1).

## Methods

### Study objectives

In this systematic review, a comprehensive literature search was conducted to identify studies investigating opioid peptide analyses in children with ASD and gluten-free casein-free (GFCF) dietary interventions in relation to opioid peptides. This systematic review had two primary objectives: (1) To assess opioid peptide concentrations in individuals with ASD compared to typically developing controls (Table 1) (2) To evaluate the efficacy of GFCF dietary interventions on opioid peptide levels and ASD symptoms (Table 2).

**Table 1:** Studies investigating opioid peptides in children with autism spectrum disorder

Study	Sample	Age (years)	Analysis method	Results
Pedersen et al. 1999 *	ASD = 135 HC = 126	-	HPLC	Close to 60% of the autistic children had an increased HPLC peak eluting like this peptide in their urine compared with controls.
Solaas et al. 2002 **	RS *n* = 53, IA *n* = 35, HC *n* = 53	2–45	HPLC	Significantly higher urinary opioid peptide concentrations were detected in individuals with Rett Syndrome.
Hunter et al. 2003	ASD *n* = 10, Twin siblings *n* = 10	2–10	LC-UV-MS (opioid peptide), ELISA (DPPIV)	No opioid peptides were detected in urine samples. Higher DPPIV concentrations in healthy siblings, but not statistically significant. No defects observed.
Dettmer et al. 2007	ASD *n* = 54, HC *n* = 15	2–6	LC-MS/MS	No opioid peptides were detected in urine samples.
Jarmolowska et al. 2007**	ASD *n* = 86, HC *n* = 51	3–10	ELISA	Serum opioid peptide concentration (β-casomorphin-7) was 1.6 times significantly higher in children with ASD.
Cass et al. 2008 *	ASD *n* = 65, HC *n* = 158	4–11	HPLC/MS	Differences found in urine profiles compared to control group, but not statistically significant.
Reichlet et al. 2012	Late-onset ASD *n* = 25, Neonatal ASD *n* = 45	2–13	HPLC, MS/MS, ELISA	Opioid peptides were detected in children with ASD.
Sokolov et al. 2014 **	ASD *n* = 10, HC *n* = 10	4–8	ELISA	Urinary opioid peptide (β-casomorphin-7) concentrations are strongly and positively correlated with CARS scores used to assess ASD symptoms. CARS scores of 30–37 indicate mild to moderate autism, whereas scores between 38 and 60 are classified as severe autism.
Tveiten et al. 2014 *	ASD *n* = 335	2–18	HPLC/MS-MS	Opioid peptides were detected in the urine of children with ASD.
Pusponegoro et al. 2015	ASD *n* = 159, HC *n* = 66	2–10	-	No opioid peptides were detected in any urine samples.
Bojovic et al. 2019 **	NDD *n* = 26 HC *n* = 26	2–9	HPLC	Casomorphin concentration were increased in 61.5% of NDD patients compared to 19.2% of healthy children. Gluten exorphins were increased in 30.8% of NDD patients and 0% of healthy children. In particu-lar, the significant increase in the concentration of CM8 and GEC was noted in NDD compared with the control group

Abbreviations: *ASD* Autism Spectrum Disorder, *CM8* β-casomorphin, 1–8, *GEC* Gluten exorphin C *HC* Healthy Controls, *RS* Rett Syndrome, *IA* Infantile Autism, *ELISA* Enzyme-Linked Immunosorbent Assay, *HPLC* High-Performance Liquid Chromatography, *LC* Liquid Chromatography, *MS* Mass Spectrometry, *NDD* Neurodevelopmental Disorders *UV* Ultraviolet, *DPPIV* Dipeptidyl Peptidase IV, *CARS* Childhood Autism Rating Scale, -: Not reported.*; higher opioid peptide concentrations.** statistically significantly higher concentrations

**Table 2:** Studies on GFCF dietary interventions and opioid peptide analysis in autism spectrum disorder

Study	Study design	Sample	Age (years)	Diet	Results
Knivsberg et al. 1990 Knivsberg et al. 1995	Follow-up	15	10–14 (girls), 6–22 (boys)	GFCF diet, 1 year & 4 years	During the follow-up period of the study, normalization in urine patterns and peptide concentrations was observed in those following the GFCF diet. Similarly, a reduction in odd behaviors and an improvement in the use of social, cognitive, and communicative skills were recorded.
Whiteley 1999	Follow-up	22	4–9	5 months GF	No significant decrease observed in urinary peptides.
Elder et al. 2006	Randomized, double-blind, crossover	15	2–16	6 weeks GFCF, 6 weeks ND	No significant differences in autism rating scores or urinary opioid peptide concentrations between diet phases.
Gonzalez-Domenech et al. 2020	Randomized, crossover	37	2–18	6 months GFCF, 6 months ND	Significant change in autism rating scores in group that started with ND and switched to GFCF. Decrease in urinary β-casomorphin, but not statistically significant.
Bavykina et al. 2021	Case-control	85	3–15	6 months GFCF	Glidiamorphin and casomorphin concentrations were significantly lower in the intervention group.

Abbreviations: *ASD* Autism Spectrum Disorder, *GFCF* Gluten-Free Casein-Free Diet, *GF* Gluten-free, *ND* Normal Diet

### Information sources and search strategy

Two independent reviewers searched PubMed, Web of Science, and Scopus databases, covering the period from January 1980 to March 2025. The search strategy combined standardized keywords and Medical Subject Headings (MeSH) terms. The search strategy included a combination of keywords commonly used in the literature to represent ASD, opioid peptides and GFCF diets. The keywords were as follows: “Autism,” “Autistic,” “autism spectrum disorder,” “ASD,” “Childhood Disintegrative Disorder,” AND “Gluten Free Diet,” “Casein Free Diet,” “Gluten Free Casein Free Diet,” “GFCF diet,” “dietary intervention” AND “opioid peptides,” “exorphins,” “beta-casomorphin,” “gluteomorphin,” “casomorphin,” “urinary peptides,” “serum peptides,” “plasma peptides,” “peptide assay,” “ELISA,” “peptide analysis”. The choice of terms was based on standardized and widely accepted definitions in relevant fields.

### Study selection

This review aimed to synthesize evidence from clinical trials examining the role of opioid peptides in ASD and the potential impact of GFCF dietary interventions on opioid peptide concentrations in individuals with ASD. Inclusion criteria for opioid peptide studies (Table 1) required that the study measured opioid peptide levels—such as exorphins, casomorphins, or gluteomorphins—in biological samples including urine, blood, or cerebrospinal fluid. Eligible studies included participants with a diagnosis of ASD or related neurodevelopmental disorders, provided a control or comparison group, reported quantitative peptide measurements, and used an observational design such as case-control, cross-sectional, or cohort. For dietary intervention studies (Table 2), inclusion criteria required randomized controlled trials (RCTs) or controlled clinical trials involving participants with a diagnosis of ASD. The intervention had to be a GFCF diet, and studies were required to assess either opioid peptide levels or ASD-related symptoms and include a control or comparison group. Exclusion criteria included animal studies; non-empirical publications such as systematic reviews, meta-analyses, editorials, and opinion pieces; case reports or case series with fewer than ten participants; studies lacking a control or comparison group; studies that did not provide sufficient methodological details for quality assessment; and non-English publications for which no translation was available.

**Fig. 2:**
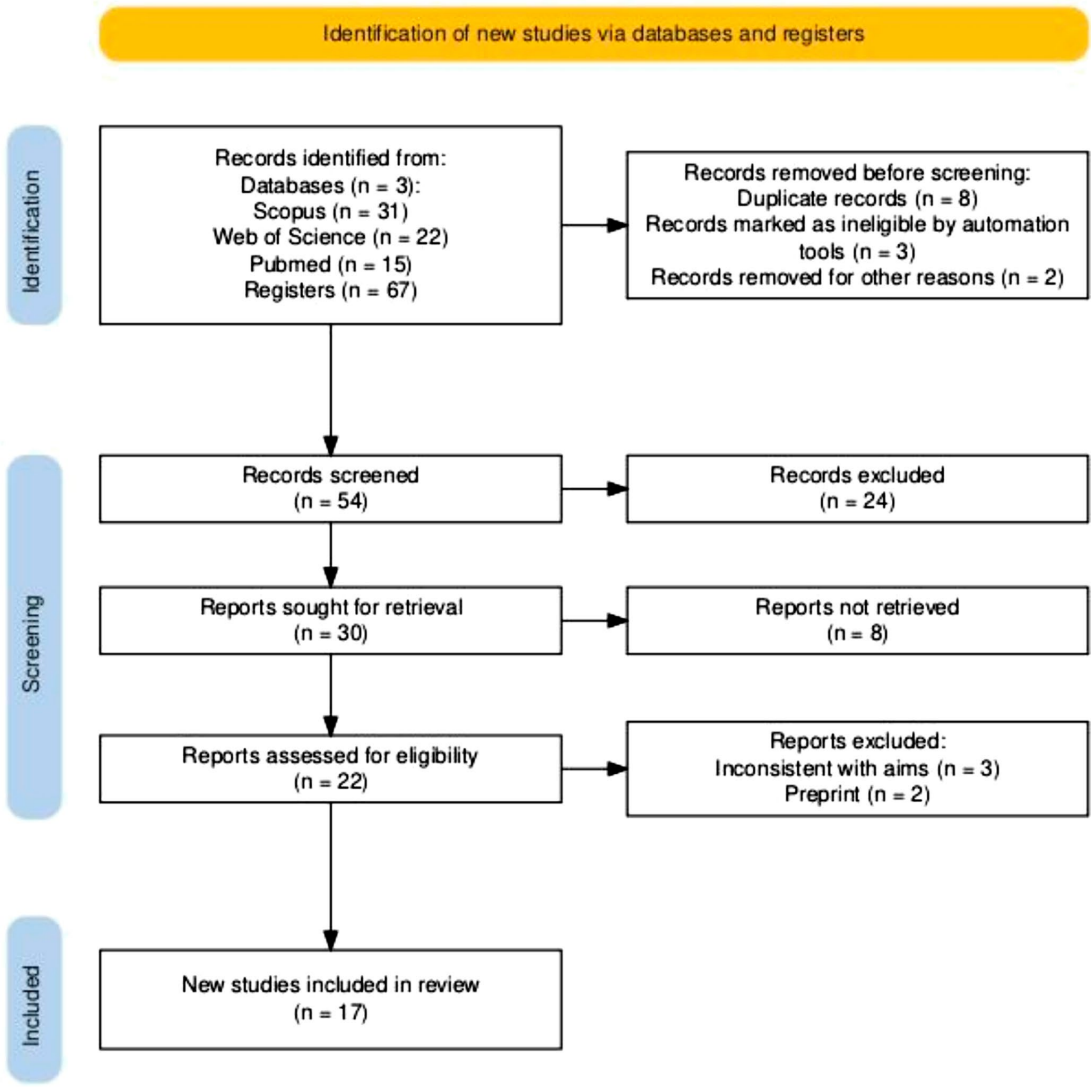
PRISMA flowchart for this review

The articles were initially selected based on an analysis of their titles and abstracts. Subsequently, a comprehensive review of the full texts was conducted The study selection process was documented in accordance with the PRISMA 2020 guidelines (Page et al. 2021). The PRISMA 2020 flow diagram was generated using the PRISMA2020 R package and Shiny app (Haddaway et al. 2022) and subsequently adapted by the authors to reflect the study-specific screening and selection steps. (Fig. 2).

### Data extraction

Data extraction was conducted independently by two reviewers using standardized forms, and any disagreements were resolved through discussion or, when necessary, consultation with a third reviewer. For the peptide studies (Table 1, *n* = 11), extracted information included study characteristics such as author, year, country, and design; participant characteristics including sample size, age, and diagnostic criteria; peptide measurement methods (HPLC, LC-MS/MS, ELISA, RIA); the types of peptides analyzed and their detection rates; and quantitative outcomes such as means, standard deviations, p-values, and confidence intervals. For the dietary intervention studies (Table 2, *n* = 6 publications representing 5 independent trials), data extraction included study design and quality indicators, sample characteristics and diagnostic criteria, details of the intervention such as diet type, duration, and adherence monitoring, outcome measures including opioid peptide levels and behavioral assessments, and reported results along with effect sizes. Knivsberg et al. (1990) and Knivsberg et al. (1995) were treated as baseline and 4-year follow-up publications from the same cohort.

### Quality assessment

The methodological quality of randomized controlled trials (*n* = 4) was assessed independently by two reviewers using the Cochrane Risk of Bias tool version 2 (RoB 2). Disagreements were resolved through discussion or consultation with a third reviewer. Risk of bias assessment results were visualized using the robvis tool (Risk-of-Bias Visualization tool). Domains assessed included randomization process, allocation concealment, blinding of participants and personnel, blinding of outcome assessment, incomplete outcome data, selective reporting, and other sources of bias. One observational study examining dietary interventions (Bavykina et al. 2021) was included in Table 2 for comprehensive reporting of diet-related studies, but formal Cochrane RoB 2 assessment was not applicable due to its case-control design. For observational studies in Table 1 (*n* = 11), formal standardized risk of bias assessment was not conducted due to substantial heterogeneity in study designs, participant characteristics, laboratory methods, and outcomes measured.

Overall quality and certainty of evidence was evaluated considering multiple dimensions adapted from GRADE (Grading of Recommendations Assessment, Development and Evaluation) methodology. For RCTs, this included assessment of: (1) risk of bias (via Cochrane RoB 2 tool), (2) inconsistency of results across studies, (3) indirectness of evidence, (4) imprecision due to sample size or confidence intervals, and (5) potential publication bias. For observational studies, quality evaluation focused on study design appropriateness, methodological rigor, sample size adequacy, and consistency of findings. Given substantial heterogeneity in study designs, populations, and outcomes, formal GRADE assessment with numerical ratings was not feasible; instead, a structured narrative synthesis of evidence quality is provided in the Results section. Overall certainty of evidence was characterized as high, moderate, low, or very low based on these considerations.

## Results

**Fig. 3:**
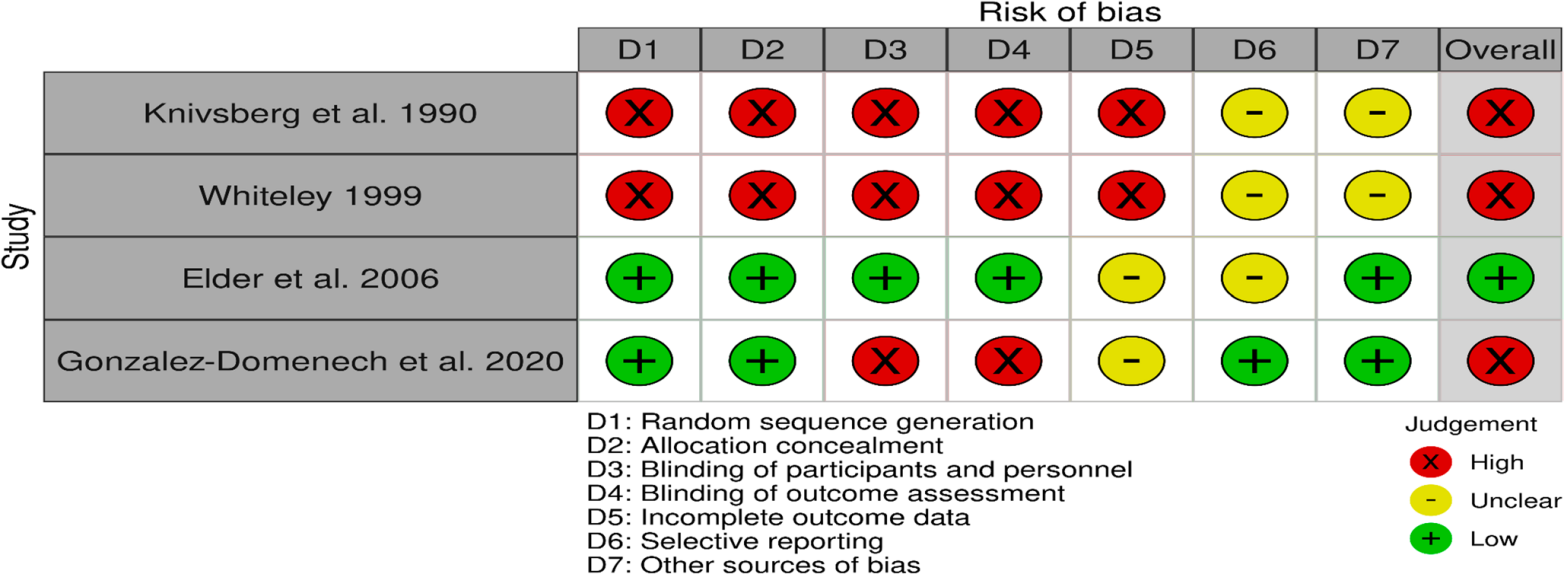
Risk of bias per study graph: review authors’ judgements about each risk of bias item for each included study. + indicates low risk of bias, X indicates a high risk of bias, and- indicates an unclear risk of bias

Studies were repetitive, resulting in a total of 54 articles. Studies on animals (*n* = 3), Reviews (*n* = 4), and unrelated studies (*n* = 1) were excluded according to title and abstracts. The number of reports assessed for eligibility is 22. The three reports that did not match the type of aims were removed. Two studies were in the preprint. Of this, 17 records were finally included in the review. These included: 11 opioid peptide studies (Table 1) and 6 publications on dietary interventions (Table 2), including one cohort with longitudinal follow-up data (Knivsberg et al. 1990; 1995). Risk of bias assessment was completed for the four randomized controlled trials examining GFCF dietary interventions (Fig. 3). One additional study in Table 2 (Bavykina et al. 2021) was a case-control observational study and therefore the Cochrane RoB 2 tool was not applicable; methodological considerations for this study are discussed separately.

Study characteristics are detailed in Tables 1 and 2. For peptide studies, sample sizes ranged from 20 to 335 participants, with ages spanning 2 to 45 years. Laboratory methods included HPLC (*n* = 4), LC-MS/MS (*n* = 3), ELISA (*n* = 3), and LC-UV-MS (*n* = 1). For dietary intervention studies, sample sizes ranged from 15 to 85 participants, with intervention durations from 5 months to 4 years.

## Autism spectrum disorder and opioid peptides

### Autism spectrum disorder

ASD was initially incorporated into the Diagnostic and Statistical Manual of Mental Disorders (DSM-III). In the DSM-IV, the disorder was classified within the broader category of pervasive developmental disorders, with specific diagnostic criteria involving social, communicative, and behavioral impairments. In the latest iteration, DSM-V, the term “autism spectrum disorder” is used as a comprehensive diagnosis that encompasses the full spectrum of these related disorders (DSM-5, 2013).

The World Health Organization (WHO) estimates that approximately 1 in 100 children is affected by ASD, with diagnosis typically occurring within the first five years of life (WHO 2023). Historical data from the CDC demonstrates an increasing prevalence of ASD over time. In 2000, the prevalence was reported as 1 in 150 children, which progressively decreased to 1 in 125 in 2004, 1 in 110 in 2006, 1 in 88 in 2008, 1 in 68 in 2010 and 2012, 1 in 59 in 2014an 2018, and 1 in 36 in 2023 in the U.S. (Baio et al. 2018). However, data from the CDC in 2025 indicates a shift, with the prevalence now estimated at 1 in 31 children in the U.S., reflecting a slight decrease in the rate of diagnosis (Shaw et al. 2025).

Currently, the primary diagnostic methods for ASD are based on developmental processes and behavioral observations, as validated and widely accepted laboratory tests are not yet available. However, particularly through chromatographic techniques, the metabolic profiles of biofluids in individuals diagnosed with ASD can be analysed. As with many other disorders, factors such as nutritional status, neurotransmitter metabolites, oxidative stress, and environmental influences affect the metabolic profile in ASD (Zurawicz et al. 2013). Biofluids, including blood, plasma, urine, and cerebrospinal fluid, can be analysed to assess these metabolic changes (Zurawicz et al. 2013). Research focused on identifying metabolic alterations in biofluids in ASD aims to facilitate better-targeted treatments and provide the potential for simpler and more objective diagnostic methods.

### Opioid peptides

Dietary intake of peptides, including gluten, casein, and gliadin, may exert opioid-like effects due to incomplete degradation in the gastrointestinal system. Casein can be converted into caseomorphin, gluten into gluteomorphin, and gliadin into gliadinmorphin. Six neurologically active peptides have been identified: beta-casomorphin, alpha-gliadin, dermorphin, deltorphin 1, deltorphin 2, and peptides modulated by morphine, including beta-casomorphin and alpha-gliadin (Hunter et al. 2003). These opioid-like peptides have been detected in various biofluids, such as serum, urine, and cerebrospinal fluid. It is well-established that these peptides originate from food including gluten-containing foods and dairy products. Food-derived opioid peptides, specifically caseomorphin from dairy and gliadorphins from gluten, originate from cereal and dairy diets. Gluten, a protein found in cereals, is primarily sourced from wheat, barley, and rye. Casein, a protein found in milk, is present in dairy products. These opioid receptors are distributed in central and peripheral nervous systems, gastrointestinal tract, immune cells, and some other tissues. These exorphins possess all the structural features that are critical for binding these opioid receptors and in turn manifestation of opioid activity (Ul Haq 2020). These peptides can affect health if they breach the intestinal barrier and interact with opioid receptors throughout the body, as they can influence various biological processes (Woodford 2021).

### Potential mechanism of opioid peptides

G protein-coupled receptors (GPCRs) are one of the four major classes of receptors embedded within the cell membrane, alongside ion channels, tyrosine kinase-coupled receptors, and intracellular receptors. GPCRs play a crucial role in mediating cellular responses to a wide variety of extracellular signals, including neurotransmitter molecules and hormones, which activate G proteins. Opioid receptors, including the µ, δ, and κ subtypes, are members of the GPCR family. Additionally, numerous pharmacological agents, such as pain relievers, antihistamines, and antipsychotic medications, primarily target GPCRs (Rueda-Ruzafa et al. 2020).

Opioid systems play a crucial role in the regulation of pain, with exogenous ligands of opioid receptors being utilized as analgesics for centuries (Pellissier et al. 2018). However, prolonged use of painkillers has led to the development of opioid addiction, which has been linked to the reward processes, one of the primary functions of opioids (Pellissier et al. 2018). While opioid receptors and their corresponding genes are involved in mechanisms related to pain and reward pathways (Le Merrer et al. 2009), they are also implicated in a variety of other physiological processes, including stress response, respiration, food intake, gastrointestinal motility, as well as endocrine and immune responses (Le Merrer et al. 2009; Pellissier et al. 2018; Sobczak et al. 2014; Yamanaka and Sadikot 2013). Specifically, opioid receptors located in the brain’s reward center, the nucleus accumbens, play a significant role in modulating social behaviors (Ragen et al. 2013). Experimental studies in animal models have demonstrated that disruption of opioid receptor-mediated neurotransmission leads to a reduction in social behaviors (Ragen et al. 2013). Moreover, administration of µ-opioid receptor agonists in both acute and chronic doses has been shown to impair long-term social memory (Trezza et al. 2011; Yim et al. 2006). In genetically modified mice lacking µ-opioid receptors, marked alterations in social behavior were observed, including reduced vocalization and diminished social interaction following maternal separation (Cinque et al. 2012). These findings suggest that µ-opioid peptide receptors are integral to the regulation of social behaviors (De Noni et al. 2009; Cinque et al. 2012; Pellissier et al. 2018; Trezza et al. 2011; Yim et al. 2006).

In addition to acting on opioid receptors, opioid peptides have also been shown to interact with toll-like receptors (TLR), particularly TLR4 (Eidson and Murphy 2013; Woodford 2021). Furthermore, casomorphins have been shown to directly affect the serotonergic system, independent of opioid receptor activation (Woodford 2021).

### Opioid excess theory

One of the theories proposed in the pathogenesis of ASD is the opioid excess theory (Tarnowska et al. 2023). This theory posits that opioid peptides cross the blood-brain barrier, interact with opioid receptors, and subsequently influence the central nervous system. It is suggested that the increased production and absorption of opioid peptides, which may occur due to enhanced intestinal permeability in individuals with ASD, is linked to the progression of the disorder. The hypothesis was first introduced by Panksepp, who proposed that morphine could play a role in the development of ASD (Panksepp 1979). A fundamental hypothesis in the etiology of ASD involves an elevation in β-endorphin, an endogenous opioid peptide. Increased concentrations of endorphins have been observed in both cerebrospinal fluid and peripheral blood in children with ASD. Furthermore, ASD is known to have a genetic component, with an inheritance rate of 50–80% in twin siblings, and increased opioid peptide concentration have been documented in the mothers of children diagnosed with ASD (Colvert et al. 2015; Garvin and Kainer 2023). Similarly, a twin study reported autism heritability rates of 96% in childhood and 87% in adulthood. (Martini et al. 2024)

Opioid peptides do not easily cross the blood-brain barrier in healthy adults. However, some may influence the brain indirectly, or may pass under certain conditions. However, certain special situations, such as immature blood-brain barrier (BBB) in infants, compromised BBB, high-dose exposure, small molecular weight, few aromatic amino acids, light hydrophobicity, light cationic properties, transport mechanisms, and indirect effects, can affect BBB transitions (Domenger et al. 2018; Maggioni et al. 2016).

The primary potential mechanism involves the incomplete degradation of casein and gluten, leading to the formation of opioid peptides that are capable of crossing the BBB. Once these peptides pass through the BBB, they are thought to interact with opioid peptide receptors, thereby influencing neurotransmission (Pellissier et al. 2018; Tarnowska et al. 2023).

Another theory thought to be associated with the development of ASD is the “leaky gut hypothesis.” This hypothesis posits that increased intestinal permeability in children with ASD may lead to enhanced absorption of agents within the gut lumen, which could contribute to the development of the disorder. The leaky gut hypothesis is often linked to the formation of opioid peptides and their subsequent passage through the blood-brain barrier, with the idea that this process may be exacerbated by heightened intestinal permeability. Furthermore, given that gastrointestinal permeability, motility, and secretion can be altered in neurological disorders, dietary interventions are aimed at modifying these changes. Opioid peptide analysis has been conducted on biological samples such as plasma, cerebrospinal fluid, and urine from children with ASD (Pellissier et al. 2018). Some studies have reported increased concentrations of opioid peptides (Jarmołowska et al. 2007), while others have found decreased concentration (Pellissier et al. 2018).

### Dipeptidyl peptidase IV defect

One of the proposed mechanisms associated with ASD is a defect in dipeptidyl peptidase IV (DPPIV) (Hunter et al. 2003). The DPPIV enzyme defect is linked to abnormal peptide content in the urine of children with autism and is believed to be associated with the disorder. DPPIV is found on the brush borders of the intestine, kidneys, and liver, on T-cell surfaces, and in certain other hematopoietic-derived cells known as CD26. Additionally, it is present in a soluble form in the blood (Reichelt et al. 1970). DPPIV plays a crucial role in the breakdown of small peptides into di- and tri-peptides that can be transported in the intestine (Reichelt et al. 1970). The degradation of these exogenous peptides results in the inactivation of opioid activities (Wallace and McKain 1997). The combination of leaky gut syndrome and DPPIV enzyme defect may lead to neurological consequences due to the presence of biologically active peptides circulating in the bloodstream (Hunter et al. 2003).

### Opioid peptide levels in ASD

Eleven observational studies examined opioid peptide concentrations in biological samples from individuals with ASD or related neurodevelopmental disorders (Table 1). Results were highly inconsistent across studies. Four studies (36%) reported elevated opioid peptide levels (Pedersen et al. 1999; Solaas et al. 2002; Jarmolowska et al. (2007); Sokolov et al. 2014). Two studies (18%) reported detection of opioid peptides without statistical comparison (Reichelt et al. 2012; Tveiten et al. 2014). Two studies (18%) reported differences in opioid peptide levels that were not statistically significant (Cass et al. 2008; Bojović et al. 2019). Three studies (27%) detected no opioid peptides (Hunter et al. 2003; Dettmer et al. 2007; Pusponegoro et al. 2015).

Pedersen et al. (1999) examined urine samples from 135 children with ASD and 126 healthy controls using HPLC. Approximately 60% of children with autism demonstrated increased HPLC peaks consistent with opioid peptides compared to controls, representing one of the largest studies suggesting peptide elevation. Solaas et al. (2002) investigated urinary opioid peptides in individuals with Rett Syndrome (*n* = 53), infantile autism (*n* = 35), and healthy controls (*n* = 53), aged 2–45 years, using HPLC. Significantly higher urinary opioid peptide concentrations were detected in Rett Syndrome compared to both autism and control groups. Jarmolowska et al. (2007) measured serum β-casomorphin-7 using ELISA in 86 children with ASD (aged 3–10 years) versus 51 controls. Serum opioid peptide concentration was 1.6-fold significantly higher in children with ASD (*p* < 0.05). Sokolov et al. (2014) examined urinary opioid peptides using ELISA in 10 children with ASD (aged 4–8 years) versus 10 controls. Urinary peptide concentrations were significantly higher in ASD and demonstrated strong positive correlation with CARS symptom severity scores.

Reichelt et al. (2012) examined children with late-onset ASD (*n* = 25) and neonatal ASD (*n* = 45), aged 2–13 years, using HPLC, MS/MS, and ELISA. Opioid peptides were detected in children with ASD, but no control group was included for statistical comparison. Tveiten et al. (2014) conducted the largest single study (*n* = 335 children with ASD, aged 2–18 years) using HPLC/MS-MS. Opioid peptides were detected in urine, but the absence of a control group precluded determination of whether levels were elevated compared to typically developing children.

Cass et al. (2008) examined 65 children with ASD (aged 4–11 years) versus 158 controls using HPLC/MS. While differences in urinary peptide profiles were observed, these did not reach statistical significance despite the relatively large control group. Bojovic et al. (2019) investigated children with neurodevelopmental disorders (*n* = 26) versus 26 controls (aged 2–9 years) using HPLC. Casomorphin was elevated in 61.5% of NDD patients versus 19.2% of controls, and gluten exorphins in 30.8% versus 0%. However, statistical significance testing was not clearly reported.

Hunter et al. (2003) examined 10 children with ASD and their 10 twin siblings (aged 2–10 years) using LC-UV-MS for peptides and ELISA for DPPIV activity. No opioid peptides were detected in any samples from either group. DPPIV enzyme activity was higher in healthy siblings but not significantly different, and no enzyme defects were observed. Dettmer et al. (2007) employed LC-MS/MS to analyze urine from 54 children with ASD (aged 2–6 years) versus 15 controls. Despite the sensitive analytical method and relatively large sample, no opioid peptides were detected in any samples. Pusponegoro et al. (2015) examined the largest negative study sample: 159 children with ASD (aged 2–10 years) versus 66 controls. No opioid peptides were detected in any urine samples. The analytical method was not specified, limiting methodological evaluation.

The overall quality of evidence regarding opioid peptide levels in ASD is rated as very low. The evidence base demonstrates marked inconsistency, with approximately 36% of studies (4/11) reporting elevated peptide concentrations, 27% (3/11) detecting no peptides in either group, and the remainder reporting non-significant or statistically uncompared findings. This heterogeneity could not be attributed to sample size alone, as both large and small investigations contributed to conflicting results. Multiple methodological limitations compromise evidence quality, including absence of blinding during laboratory analysis, evolution of diagnostic criteria across two decades, substantial variability in sample handling protocols, limited statistical power, and infrequent confidence interval reporting. Most investigations measured urinary peptides without establishing clinical relevance; only Sokolov et al. (2014) examined symptom correlations. Critically, a systematic pattern emerged whereby earlier studies employing less specific HPLC methods (Pedersen et al. 1999; Solaas et al. 2002) more frequently reported detection, whereas investigations utilizing high-specificity LC-MS/MS (Dettmer et al. 2007; Hunter et al. 2003) often detected no peptides, raising concerns regarding false-positive findings in earlier research. Current evidence cannot determine whether peptide alterations are truly absent, heterogeneously distributed, or detectable only under specific conditions. Future research employing standardized, high-specificity analytical methods with assessment of clinical correlates is essential.

### GFCF dietary interventions in ASD

Four RCTs have examined GFCF dietary interventions in children with ASD (Table 2). Knivsberg et al. (1990, 1995) followed 15 children aged 6–22 years on a GFCF diet for one year, with a subsequent 4-year follow-up. The initial report indicated normalization of urinary peptide patterns and improvements in behavioral problems, while the follow-up study reported sustained gains in communication and social behaviors. However, the open-label design and reliance on non-blinded parental ratings limit the interpretability of these findings. Whiteley (1999) conducted a follow-up study with 22 children aged 4–9 years over a 5-month GFCF diet period, observing no significant decrease in urinary peptides; the open-label design and lack of adequate randomization reduce confidence in these results. Elder et al. (2006) performed a double-blind crossover RCT with 15 children aged 2–16 years, comparing 6-week periods of GFCF and regular diets with an appropriate washout. No significant differences were found in autism rating scores or urinary opioid peptide concentrations, and this was the only study with adequate blinding across all domains. Gonzalez-Domenech et al. (2020) conducted a crossover RCT with 37 children aged 2–18 years, comparing 6-month periods of GFCF and regular diets. Significant behavioral improvements were observed in the group starting with a regular diet and switching to GFCF, but urinary β-casomorphin decreased non-significantly, and inadequate blinding of outcome assessors limits confidence in the results.

**Fig. 4:**
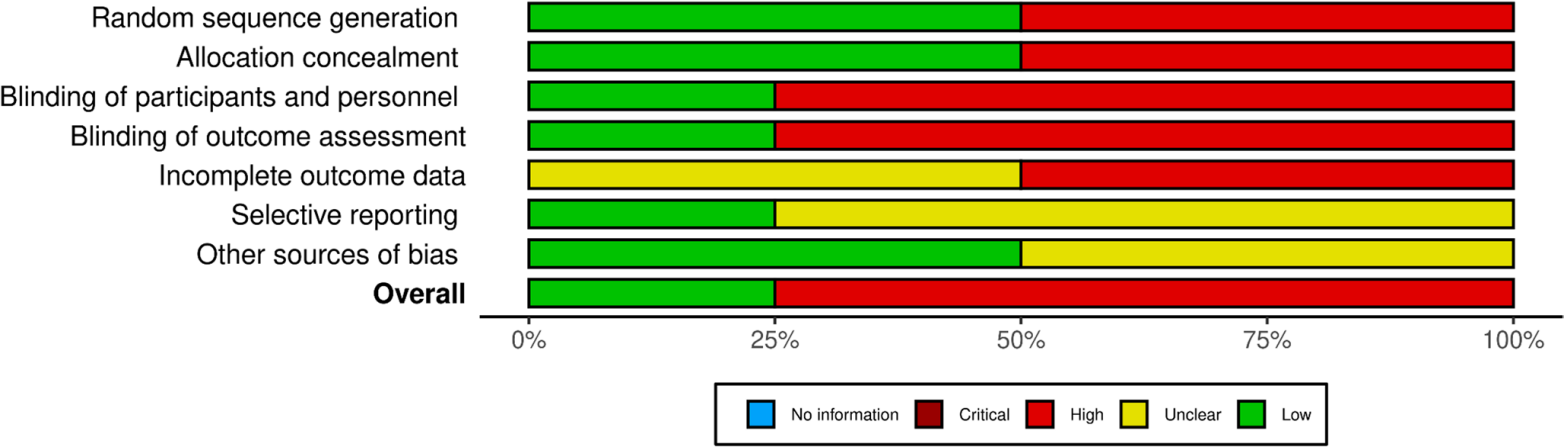
Risk of bias summary graph: review authors’ judgements about each risk of bias item presented as percentages across all included studies

Risk of bias assessment was completed for four RCTs examining GFCF dietary interventions (Figs. 3 and 4). Only Elder (2006) demonstrated low overall risk of bias (25%), while three studies (75%) showed high overall risk. Across 28 domain-level assessments, 54% were rated high risk, 32% unclear risk, and only 14% low risk. Methodological weaknesses were concentrated in several domains. Randomization and allocation concealment were adequate in only 50% of studies (Elder 2006, Gonzalez-Domenech 2020), with Knivsberg (1990) and Whiteley (1999) providing unclear descriptions. Blinding represented the most serious limitation: 75% of studies failed to adequately blind participants and personnel, and 75% failed to blind outcome assessors. Critically, three studies (Knivsberg 1990, Whiteley 1999, Gonzalez-Domenech 2020) relied on non-blinded parental behavioral reports as primary outcomes, creating substantial risk of expectancy and detection bias. Only Elder (2006) successfully implemented double-blind procedures throughout. Incomplete outcome data was problematic in 75% of studies, with Knivsberg (1990) and Whiteley (1999) showing high risk due to substantial attrition and incomplete reporting, while Elder (2006) and Gonzalez-Domenech (2020) received unclear ratings due to insufficient detail. Selective reporting assessment was challenging, as 75% of studies lacked available protocols; only Gonzalez-Domenech (2020) demonstrated comprehensive pre-specified outcome reporting. Other sources of bias represented the best-performing domain, with 75% rated as low risk. The observational study (Bavykina et al. 2021) was not assessed using Cochrane RoB 2 and is not represented in Figs. 3 and 4. This study has critical methodological limitations including selection bias, cross-sectional design precluding causality, variable unmonitored diet duration, lack of adherence verification, no control for concurrent therapies, and no blinding of laboratory analyses, resulting in very low confidence in findings.

## Discussion

The approach of eliminating gluten and casein as a therapeutic dietary measure for ASD development has become increasingly popular among families. This elimination diet involves removing gluten- and casein-containing foods to reduce the production of exogenous opioid peptides and lower their concentration in biofluids. Research indicates that approximately 25% of families with children diagnosed with ASD adopt a GFCF diet (Reissmann et al. 2014; Whiteley et al. 2013). Furthermore, in a systematic review, this rate was reported to range from 8% to 32% (Reissmann 2020).

There are many studies investigating the presence of opioid peptides in the urine and serum of children diagnosed with ASD (Table 1). Some studies have demonstrated that these peptides are increased in individuals with ASD compared to control groups (Bojović et al. 2019; Cass et al. 2008; Jarmołowska et al. 2007; Pedersen et al. 1999; Sokolov et al. 2014; Solaas et al. 2002; Tveiten et al. 2014). Furthermore, the concentration of urinary opioid peptides has been linked to the severity of behavioral issues in these children (Sokolov et al. 2014). Sokolov et al. reported a positive correlation between total opioid peptide concentrations and CARS scores. They found that as opioid peptide concentration increase, CARS scores reflecting the severity of ASD symptoms also increase. This correlation was evaluated using the total CARS score; however, the association was not analyzed separately for the 15 sub-items of the scale (Sokolov et al. 2014). Contrary to these studies, some studies cannot detect opioid peptides (Dettmer et al. 2007; Hunter et al. 2003; Pusponegoro et al. 2015).

Studies investigating the relationship between opioid peptide concentrations and the potential therapeutic effects of dietary interventions, particularly GFCF diets, in individuals with ASD remain relatively limited (Table 2) (Bavykina et al. 2021; Elder et al. 2006; González-Domenech et al. 2020; A.-M. Knivsberg et al. 1990; Ann-Mari Knivsberg et al. 1995; Whiteley et al. 1999). Among the earliest research in this area were studies conducted by the Knivsberg and Reichelt research groups (A.-M. Knivsberg et al. 1990; Ann‐Mari Knivsberg et al. 1995). These investigations demonstrated a reduction in urinary peptide concentrations associated with gluten and casein metabolism following dietary intervention. In particular, concentrations of the opioid peptide beta-casomorphin were found to be significantly correlated with age. Although a decline in mean beta-casomorphin concentrations was observed after implementation of a GFCF diet, this reduction did not reach statistical significance.

In studies where dietary intervention through a gluten-free and/or casein-free diet was implemented, limited but suggestive evidence has indicated that such interventions may exert a modest positive effect on certain parameters associated with ASD development (Milward et al. 2018). Research examining the relationship between gluten-free diets and opioid peptide concentrations has shown that individuals adhering to such diets tend to exhibit lower concentrations of blood opioid peptides compared to those following a standard diet (Bavykina et al. 2021; González-Domenech et al. 2020). Notably, these reductions were found to be statistically significant in some studies (Bavykina et al. 2021). However, conflicting findings exist in the literature, with several studies reporting no significant differences in opioid peptide concentrations or ASD symptomatology between individuals adhering to a GFCF diet and those in control groups (Elder et al. 2006; González-Domenech et al. 2020; Reissmann et al. 2014; Whiteley et al. 1999).

In children diagnosed with ASD, GFCF dietary interventions may yield improvements in primary and secondary symptoms, even in the absence of direct assessment of opioid peptide concentrations. For example, a study by Knivsberg et al. (2002) reported statistically significant improvements in verbal communication, attention, social and sensory responsiveness, and general communication and skill scores among children receiving dietary intervention, compared to a control group (Knivsberg et al. 2002). Similarly, in a subsequent study by the same research group (Knivsberg et al. 2003), significant enhancements were observed in communication and social interaction metrics relative to controls (A.-M. Knivsberg et al. 2003). Whiteley et al. (2010) conducted a randomized controlled trial evaluating a GFCF diet over two 12-month phases (Whiteley et al. 2010). In the initial phase, participants in the dietary intervention group demonstrated significant improvements in social communication, attention, and hyperactivity scores compared to controls. Additionally, daily living skills among children with ASD were significantly enhanced. In the second phase of the study, further significant improvements were observed in communication, repetitive behavior, and socialization scores. Contrastingly, a double-blind, placebo-controlled study by Hyman et al. (2016) evaluated the effects of gluten-free, casein-free, gluten-only, casein-only, and placebo snack consumption, contributing to the growing but still inconclusive evidence base regarding the efficacy of dietary interventions in ASD populations. At the conclusion of the study, no statistically significant differences were found between the dietary intervention groups and the placebo group in terms of autism symptom severity, behavioral outcomes, or physiological parameters (Hyman et al. 2016).

### Current limitations

There are some limitations in the conduct of the studies. One of these concerns the analysis techniques. It is essential to highlight that the analytical techniques used - such as ELISA, HPLC, MS - are extremely sensitive. Without appropriate calibration curves, however, these methods may lack reproducibility. Additionally, exorphins in urine samples degrade rapidly, making it crucial to implement specific precautions during analysis to ensure sample stability (Reichelt et al. 2012; Tveiten et al. 2014). Therefore, accurate interpretation of findings depends not only on the validity of the research but also on the careful execution of peptide analysis protocols.

Another limitation relates to bio-fluids. Opioid peptides can be analysed in bio-fluids such as blood, plasma, and urine. However, the studies reviewed for this review generally preferred the analysis of urine samples. This may be due to the complex nature of the blood matrix, which can compromise peptide stability and complicate purification steps. In contrast, the urine matrix is cleaner and provides a more stable environment for analysis. Additionally, urine samples are easier to collect from individuals with ASD. Samples such as blood, which must be collected invasively, can be quite challenging for individuals with ASD and their caregivers.

One of the limitations of GFCF dietary interventions is the difficulty in adhering to the diet. Studies have shown that 88% of children with ASD have eating disorders, and 53% experience difficulty trying new foods (González-Domenech et al. 2020). This challenge is thought to contribute to the difficulty individuals with ASD face in adapting to the GFCF diet. As a result, the number of studies utilizing GFCF dietary interventions is limited. Additionally, the difficulty of implementing the GFCF diet may contribute to small sample sizes and short intervention durations in existing studies.

### Conclusion and recommendations

Numerous studies have been conducted using chromatographic methods examining bio-fluids and addressing the diagnosis, treatment, and progression of ASD. However, the sensitivity of analytical techniques and the rapid degradation of opioid peptides at room temperature must be considered.

Consumed nutrients are associated with the formation of some intermediate metabolites in the body. While there are studies demonstrating a correlation between dietary interventions and potential biomarkers, research in this area is insufficient. However, small sample sizes and sample heterogeneity are among the reasons for the statistically non-significant findings. Therefore, a methodologically robust randomized controlled trial design with a crossover and longer intervention duration would increase the significance and reliability of the results. Furthermore, when examining the relationship between diet and opioid peptides, the inclusion of additional assessments such as gastrointestinal symptoms, intestinal permeability measurements, intestinal bacterial populations, and gastrointestinal enzymatic and inflammatory activity would increase the power of the studies. In addition, examining gut health and intestinal structural and functional changes is also considered important.

Consequently, there is no strong evidence for any metabolite that definitively explains the etiology or pathophysiology of ASD. While some studies have suggested that GFCF diets improve autistic symptoms, others have not found significant behavioral improvements or significant reductions in urine peptide concentration. This limits the diet’s widespread recommendation for the general population and suggests that future research should focus on identifying specific ASD subgroups that may benefit from the diet. Given the unique needs and requirements of individuals diagnosed with ASD and their families, further research is needed in this area.

## Data Availability

No datasets were generated or analysed during the current study.

## Declarations

### Systematic review registration

PROSPERO CRD420251207178.
